# Twentieth Century Practice

**Published:** 1899-09

**Authors:** 


TWENTIETH CENTURY PRACTICE.—An International Encyclopedia of Modern Medical Science by leading authorities of Europe and America. Edited by Thomas L. Stedman, M. D. In twenty volumes. Volume XVII., Infectious Diseases and Malignant New Growths. William Wood & Company, Publishers. New York City.

The first subject treated of in this volume is diphtheria. Few works on medicine contain a treatise so exhaustive or so scientific on this very important subject. Dr. William Hallock Park, of New York City, discusses the general pathology and bacteriology of diphtheria, and Dr. A. Jacobi, of New York City, its symptomatology and treatment. These cover 118 pages of the book. Dr. Victor Babes, of Bucharest, has written a very interesting article on tetanus. In this paper he has given a history of the antitoxin treatment of this disease, and its status at the present time.

About 300 pages of this volume are taken up in the discussion of cancer; first, the general pathology, by Dr. W. Roger Williams, of Bristol, England, and second, the symptomatology and treatment, by Dr. William B. Coley, of New York.

The general pathology of sarcoma has been well discussed by Dr. W. Roger Williams, and its symptomatology and treatment, by Dr. Wm. B. Coley.

Malignant new growths of the skin is the subject of an article by Dr. John T. Bowen, of Boston; and malignant diseases of the female organs of generation the subject of another, by Dr. Edward McGuire, of Richmond, Va. All of the articles in this volume are written by men eminently qualified to discuss the very important subjects upon which they treat, and this volume is beyond question a valuable addition to our literature. S. E. H.

